# 289. Prolonged Corticosteroid Usage, Associated Infectious Complications, and High Mortality in Adult Patients with COVID-19

**DOI:** 10.1093/ofid/ofac492.367

**Published:** 2022-12-15

**Authors:** Rebecca Chu, Veronica B Zafonte, Kelly L Cervellione, Andrew S Miele, Farshad Bagheri, Javeria Shakil

**Affiliations:** Medisys Health Network, Jamaica, New York; Jamaica Hospital Medical Center, Jamaica, New York; MediSys Health Network, Jamaica, New York; MediSys Health Network, Jamaica, New York; Jamaica Hospital Medical Center, Jamaica, New York; Flushing Hospital Medical Center, Jamaica, New York

## Abstract

**Background:**

Based on the RECOVERY trial, the NIH strongly recommends systemic corticosteroids for COVID-19 pneumonia with hypoxia. The study demonstrated reduced 28-day mortality after dexamethasone 6mg daily for up to 10 days compared to SOC alone. In practice, physicians may continue therapy for longer periods if potential benefit outweighs potential risk. Little is known about adverse events associated with prolonged corticosteroid use in patients with COVID-19; however, in general, this can increase risk for bacterial or fungal infections. This study aimed to explore the incidence of secondary infections during extended corticosteroid use for COVID-19.

**Methods:**

A retrospective study was performed in adults at two community hospitals between September 2020 and May 2021 with a diagnosis of respiratory failure secondary to COVID-19 infection. Those with at least one new, laboratory-confirmed secondary infection during hospitalization after at least 10 cumulative days of corticosteroids were included. Demographic characteristics, clinical data and outcomes were extracted from the medical records. Quantitative analyses was performed using SPSS v27.0 and R.

**Results:**

Of over 1,500 COVID-19 admissions within the timeframe, 73 patients met inclusion criteria (**Table 1**). No patient received immunomodulators for COVID-19 treatment. Patients had a median of 18 days on corticosteroids (range 10-65 days) prior to first positive culture. There were 130 positive cultures identified in blood, urine, and sputum samples (**Figure 1**), including polymicrobical cultures, yielding 34 clinically relevant organisms (**Table 2**). Hospital course was complicated by septic shock (68.5%), worsening of lung function (76.7%), and acute organ damage (57.5%); 55 (75%) patients expired during index admission.

**Figure:**
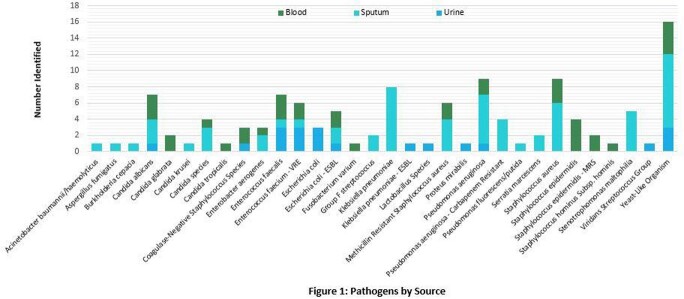

**Figure:**
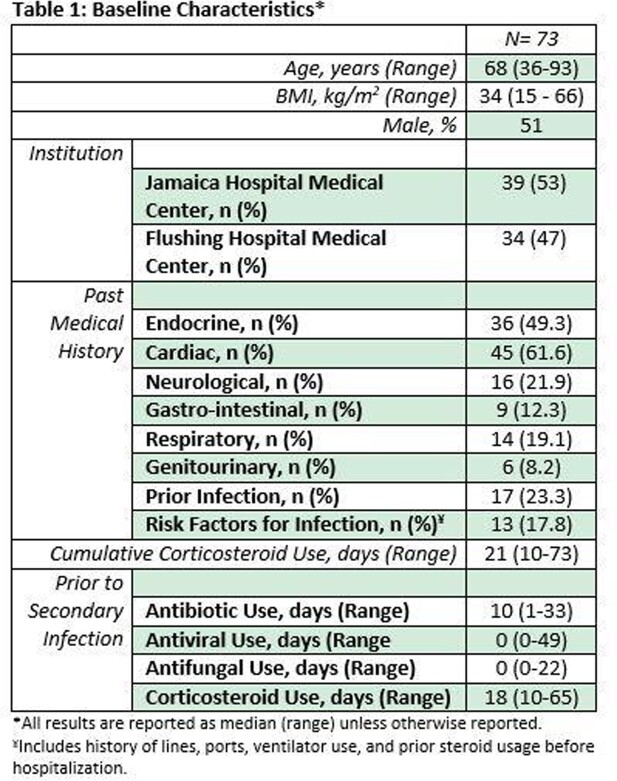

**Figure:**
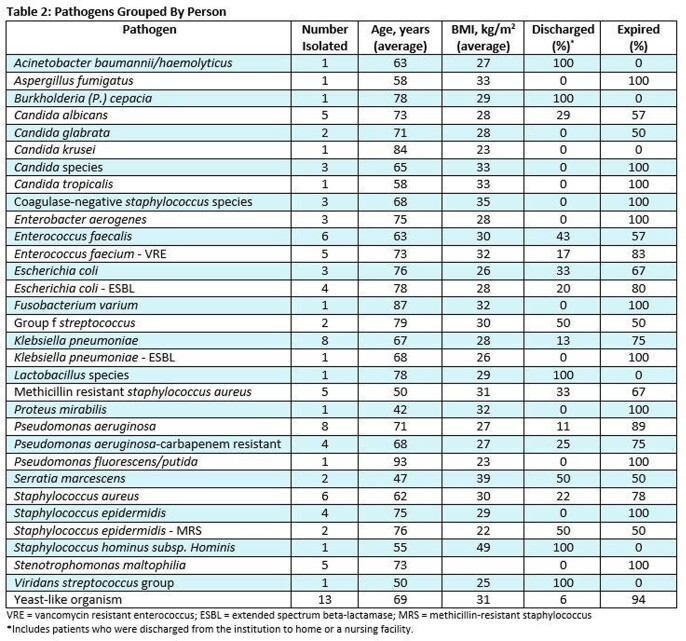

**Conclusion:**

Patients with COVID-19 on extended courses of corticosteroids have relatively higher rates of clinically significant invasive secondary infections and associated poor outcomes. Given there is limited information suggesting improved outcomes with these prolonged courses, risk to benefit analysis must be considered when deciding whether to extend corticosteroid treatment for more than 10 days for COVID-19.

**Disclosures:**

**All Authors**: No reported disclosures.

